# Dancing Out of Step: A Case of Tuberculous Meningitis Presenting as Childhood Chorea

**DOI:** 10.5334/tohm.871

**Published:** 2024-04-10

**Authors:** Jao Jarro B. Garcia, Cherie Marie A. Tecson-Delos Santos

**Affiliations:** 1 Division of Adult Neurology, Department of Neurosciences, College of Medicine and Philippine General Hospital, University of the Philippines Manila, Manila, Philippines; 2 Division of Pediatric Neurology, Department of Neurosciences, College of Medicine and Philippine General Hospital, University of the Philippines Manila, Manila, Philippines

**Keywords:** chorea, tuberculous meningitis, TB meningitis

## Abstract

**Background:**

Acute to subacute pediatric movement disorders require prompt diagnosis to identify potentially treatable diseases.

**Case Report:**

We present a 6-year-old male with a three-week history of generalized chorea transitioning to predominantly right-sided hemichorea and then to left hemiplegia.

**Discussion:**

We review the mechanisms in tuberculous meningitis underlying his movement abnormalities.

## Introduction

Acute to subacute onset pediatric movement disorders (MD) require prompt diagnosis to identify potentially treatable diseases [1,2]. Currently, there is a dearth of data regarding these conditions and there are no available large population studies determining true incidence in children [2]. Hyperkinetic MDs are more frequent in pediatrics as compared to hypokinetic MDs. Differentials for the former are dependent on the predominant phenomenology, age at presentation, time frame, and other associated manifestations [1,2,3]. We report an atypical case of subacute pediatric chorea that highlights the importance of prompt diagnosis of a treatable condition.

## Case Presentation

A 6-year-old, developmentally at par, right-handed, Filipino male was admitted in our institution because of involuntary limb movements. He had a three-week history of headache and intermittent, undocumented fever which eventually became associated with involuntary dance-like movements of all his extremities. He was initially managed at a different hospital as a case of rheumatic fever and was given a course of parenteral penicillin. The diagnosis was subsequently revised to autoimmune encephalitis and he was later on discharged on oral valproic acid syrup. In the interim, the parents noted resolution of the involuntary movements on the left but persistence of the abnormal movements on the right. This prompted consult at our institution.

Ancillary history revealed no recurrent sore throat, respiratory or gastrointestinal symptoms, easy fatigability, or joint pains. He had no deterioration in sensorium, behavioral changes, seizures, or focal neurologic deficits. He had close contact exposure to a known case of pulmonary tuberculosis (TB) but had no other known co-morbidities, previous medication intake, or prior viral exanthems. There were no family members with the same symptomatology and the rest of his ancillary history was unremarkable.

Physical examination revealed no dysmorphisms, alopecia, Kayser-Fleischer rings, anterior neck masses, or cutaneous lesions. He had brief, non-rhythmic, non-stereotyped, flowing movements predominantly on the right leg more than the right arm. Minimal choreiform movements were also appreciated on the left upper limb. Hand spooning and parakinesia were likewise observed on his right upper limb. There were no other observed abnormal movements or posturing involving other body regions. Video 1 shows the patient’s abnormal movements at the time of consult at our institution. He had full and smooth pursuit and full and quick saccades. His extremities were normotonic and had a muscle motor strength of 5. There were no upper motor neuron signs, nystagmus or dysmetria. The rest of his neurologic examination was unremarkable.

**Video 1:** Video documentation of the patient’s abnormal movements upon consult at our institution. Chorea was predominantly seen on the right leg more than the right arm. Minimal choreiform movements were also appreciated on the left upper limb. Parakinesia and spooning sign, seen as finger hyperextension interrupted by choreiform movements, were also noted on the right upper limb.

The diagnostics from the initial admission were not available for review. Baseline work-up at our institution, however, revealed elevated peripheral white blood cell (WBC) at 14.6 × 10^9^/L (Normal: 4.5–11) and low hemoglobin at 113 g/L (Normal: 135–180). Random blood glucose was 82mg/dL (Normal: 74–106) while serum sodium was 129 mmol/L (Normal: 135–145). His platelet count, creatinine, liver enzymes, serum calcium, and serum magnesium were all within normal limits. Chest X-ray revealed normal findings and anti-streptolysin O (ASO) titer was <200 IU/mL (Normal: <200). He also tested negative for COVID polymerase chain reaction (PCR).

Because of the subacute history of fever and headache plus the absence of apparent acquired causes of chorea, a lumbar puncture was performed to investigate for central nervous system (CNS) infections. Cerebrospinal fluid (CSF) studies demonstrated lymphocytic-predominant leukocytosis (WBC 144 × 10^6^/L, Lymphocytes 99%), hyperproteinorrachia (351mg/dL; Normal: 12–60), and hypoglycorrhachia (undetectable; Normal: 40–70mg/dL). CSF gram stain and culture, acid-fast bacilli (AFB), *Mycobacterium tuberculosis* PCR, and anti-N-methyl-D-aspartate receptor (NMDA-r) antibody all came back negative. Given the endemicity of TB in the Philippines, the patient’s known exposure to a confirmed case of TB, his subacute presentation, and the constellation of CSF findings, a diagnosis of Tuberculous Meningitis (TBM) British Medical Research Council (BMRC) Stage III was made and he was started on isoniazid/rifampicin/pyrazinamide/ethambutol 150/75/400/275 mg fixed-combination tablet, 2 tablets once a day and intravenous dexamethasone at 20 mg/day. Symptomatic treatment for the chorea was not started immediately. The medications for TBM was prioritized as this was the underlying cause of his movement abnormalities.

**Figure 1:**
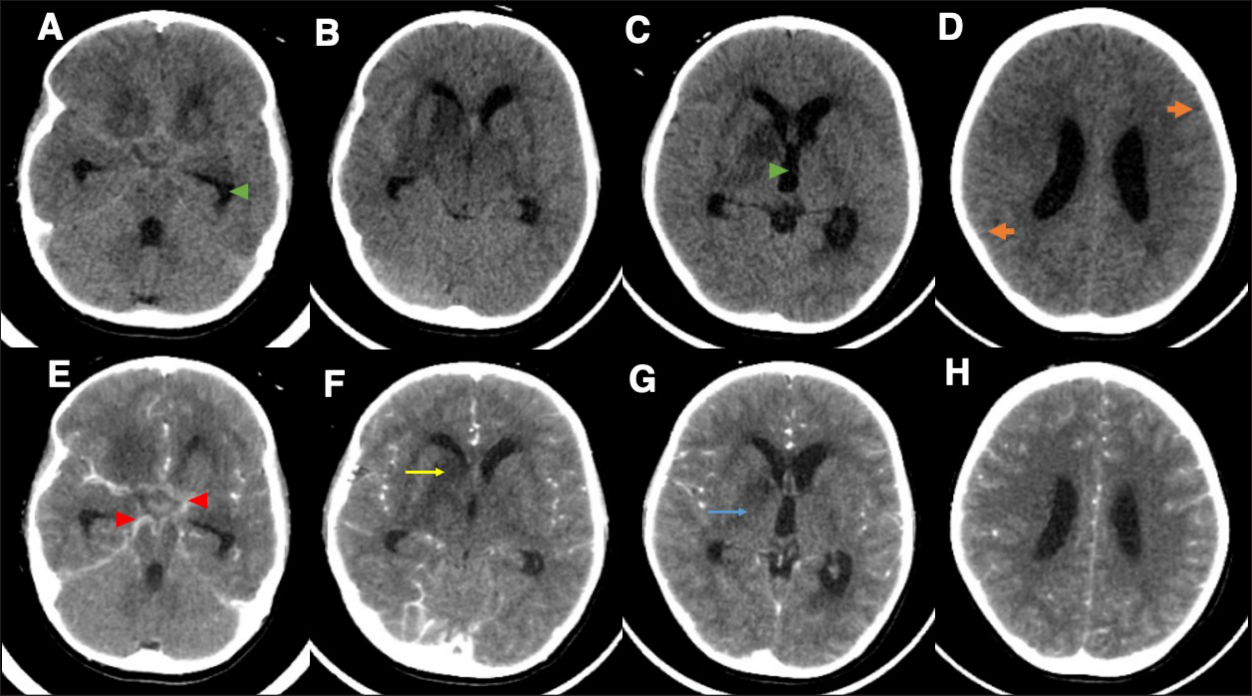
Cranial CT with Contrast. **[A-D]** Plain cranial CT demonstrating beginning communicating hydrocephalus as evidenced by the dilated temporal horns *(green arrowhead, A)* and third ventricle *(green arrowhead, C)*. Also shown is diffuse cerebral edema as evidenced by the effaced sulci-gyri interface *(thick orange arrows, D)*. **[E-H]** Contrast-enhanced sequences showing basal enhancements *(red arrowheads, E)* and non-enhancing hypodensities involving the right caudate head *(thin yellow arrow, F)*, right lentiform nucleus, & right internal capsule *(thin blue arrow, G)*. Also seen are undue leptomeningeal enhancements.

On the third day of hospitalization, his choreiform movements ceased but he was suddenly seen with new onset left-sided central facial palsy and spastic hemiplegia. Contrast-enhanced cranial computed tomography (CT) revealed basal exudates, communicating hydrocephalus, leptomeningeal enhancement, and diffuse cerebral edema. Non-enhancing hypodensities deemed to be arteritic infarcts were also noted in the right caudate head, lentiform nucleus, and internal capsule (see Figure 1). The over-all radiographic picture, particularly the triad of hydrocephalus, basal enhancements, and infarcts, supported the diagnosis of TBM [4]. Further imaging with cranial magnetic resonance imaging was not pursued because the said modality was not available at the time of his admission.

He was then started on mannitol 3cc/kg/dose to address the cerebral edema, acetazolamide 25 mg/kg/day to alleviate the communicating hydrocephalus, and aspirin 4 mg/kg/day for the arteritis. Medical therapy was maximized and decompression with mannitol was subsequently tapered until discontinued given the absence of signs of increased intracranial pressure. He was eventually sent home in stable condition.

On follow-up two weeks post-discharge, he still had left hemiplegia but there were no recurrence of the previously noted chorea. Long-term plan for the patient was to complete 1 year of anti-TB regimen, gradually taper off and discontinue his oral dexamethasone, and continue with his physical therapy sessions.

## Discussion

Chorea is an irregular, non-suppressible, dance-like movement flowing from one body part to another [1]. Etiology is typically divided into primary and secondary causes. Genetic diseases are typically implicated in the former whereas the latter are attributed to underlying CNS insults. Another classification scheme divides the etiology of chorea by time course. Acute chorea results from toxic exposures, underlying autoimmune conditions, or infectious or para-infectious causes whereas chronic chorea is associated with prior perinatal insult, familial history of the same disease, and neurobehavioral decline [1]. For the case presented, secondary causes of acute to subacute chorea were mainly considered and investigated.

Drug-induced chorea was deemed unlikely given the absence of any prior medication intake. Chorea secondary to polycythemia, hepatic failure, azotemia, hyperglycemia, or other electrolyte imbalances were also ruled out after his baseline diagnostics came out [5,6]. Hyponatremia as the cause of chorea was deemed less likely because neurologic manifestations, including choreoathetoid movements, typically manifest in severe hyponatremia when serum sodium levels are <120 mmol/L [7]. Sydenham chorea, the most common type of acute to subacute autoimmune chorea in childhood, was also considered. History often reveals repeated Group A *beta-hemolytic streptococcal* infection with or without carditis or arthritis [8]. There is also a prolonged latency from infection to the development of chorea. However, the absence of these points in his history and the normal ASO titers made the differential less likely. Another autoimmune condition considered was anti-NMDAr encephalitis. However, the negative CSF anti-NMDAr antibody result plus the absence of neuropsychiatric manifestations, seizures, and dysautonomia made this autoimmune encephalitis less considered [9]. Infectious causes were then pursued. His known exposure to a confirmed case of pulmonary TB, subacute clinical presentation, CSF findings, and neuroimaging results all supported the diagnosis of TBM despite negative CSF TB AFB and TB PCR results. It must be pointed out that bacteriologic confirmation of TB is often difficult because of the bacterium’s paucibacillary nature [4].

The prevalence of MDs among patients with TBM range from 0.5 to 18.6%. The higher the BMRC grade, the greater is one’s predisposition to develop movement abnormalities [10,11,12]. Cases manifesting with parkinsonism, tremors, dystonia, myoclonus, chorea, or hemiballismus had been documented and tremor was noted to be the most common phenomenology [4,6].

The pathomechanism of MDs in TBM is usually multifactorial. Cranial imaging may show focal structural lesions in the basal ganglia contralateral to the symptomatic side [13]. Chorea had been observed in those with caudate pathologies, parkinsonism in those with midbrain involvement, and dystonia in patients with lesions involving the globus pallidus and thalamus [14]. Some may also have diffuse lesions at the diencephalic-mesencephalic level. Ischemic strokes occurring early in TBM are a sequelae of vasospasm whereas those developing later in the course result from proliferative intimal disease [13]. There may also be instances wherein there is poor correlation between the clinical findings and the focal lesions on neuroimaging [15]. In these cases, MDs may arise from diffuse underlying pathologies such as direct neuronal injury, basal ganglia neurotransmitter dysregulation, hydrocephalus, basal exudates, and cerebral edema [15,16]. These non-localizing mechanisms likely explain why our patient initially presented with generalized chorea before it transitioned to predominantly right-sided hemichorea. The arteritis which developed in the right caudate head, lentiform nucleus, and internal capsule then caused his new onset left-sided weakness.

Our case is unusual as compared to other reported cases of TBM-associated chorea because ours demonstrated generalized chorea transitioning to predominantly right-sided hemichorea and then to left hemiplegia. These likely resulted from the gradually progressive meningitic and then vasculitic processes from the underlying TBM. Our patient’s scenario also highlights the importance of having a wide array of differentials for acute to subacute pediatric MDs. It also emphasizes the value of prompt diagnosis and intervention to prevent complications of potentially treatable conditions.

## Abbreviations

AFB – acid-fast bacilli

ASO – anti-streptolysin O

BMRC – British Medical Research Council

CNS – central nervous system

CSF – cerebrospinal fluid

CT – computed tomography

MD – movement disorders

NMDA-r – N-methyl-D-aspartate receptor

PCR – polymerase chain reaction

TB – tuberculosis

TBM – tuberculous meningitis

WBC – white blood cell
